# Effect of Annealing on the Thermoelectricity Properties of the WRe26-In_2_O_3_ Thin Film Thermocouples

**DOI:** 10.3390/mi11070664

**Published:** 2020-07-07

**Authors:** Bian Tian, Yan Liu, Zhongkai Zhang, Zhaojun Liu, Libo Zhao, Qijing Lin, Peng Shi, Qi Mao, Dejiang Lu, Zhuangde Jiang

**Affiliations:** 1 State Key Laboratory for Mechanical Manufacturing Systems Engineering, Xi’an Jiaotong University, Xi’an 710049, China; z.zhongkai@stu.xjtu.edu.cn (Z.Z.); lzj2018@stu.xjtu.edu.cn (Z.L.); libozhao@mail.xjtu.edu.cn (L.Z.); qjlin2015@mail.xjtu.edu.cn (Q.L.); mq.mq@xjtu.edu.cn (Q.M.); djlu@xjtu.edu.cn (D.L.); zdjiang@mail.xjtu.edu.cn (Z.J.); 2 Electronic Materials Research Laboratory, Key Laboratory of the Ministry of Education & International Center for Dielectric Research, Xi’an Jiaotong University, Xi’an 710049, China; spxjy@xjtu.edu.cn

**Keywords:** WRe26-In_2_O_3_ thin films, thermocouples, annealing, magnetron sputtering

## Abstract

WRe26-In_2_O_3_ (WRe26 (tungsten-26% rhenium) and In_2_O_3_ thermoelectric materials) thin film thermocouples (TFTCs) have been fabricated based on magnetron sputtering technology, which can be used in temperature measurement. Many annealing processes were studied to promote the sensitivity of WRe26-In_2_O_3_ TFTCs. The optimal annealing process of the thermocouple under this kind of RF magnetron sputtering method was proposed after analyzing the properties of In_2_O_3_ films and the thermoelectric voltage of TFTCs at different annealing processes. The calibration results showed that the WRe26-In_2_O_3_ TFTCs achieved a thermoelectric voltage of 123.6 mV at a temperature difference of 612.9 K, with a sensitivity of up to 201.6 µV/K. Also, TFTC kept a stable thermoelectric voltage output at 973 K for 20 min and at 773 K for two hours. In general, the WRe26-In_2_O_3_ TFTCs developed in this work have great potential for practical applications. In future work, we will focus on the thermoelectric stability of TFTCs at higher temperatures.

## 1. Introduction

The accurate measurement of high temperature is particularly important in modern science. With the development of MEMS technology, TFTCs are widely used in many areas [1,2,3,4]. TFTCs have many advantages, such as fast response, high measurement accuracy, and easy integration [5,6,7]. Traditionally, for metal TFTCs, Such as type-K (Ni_10_Cr/Ni_5_Si) and type-S (Pt-10%Rh/Pt) TFTCs [8,9,10,11]. This kind of metal TFTCs have low sensitivity and low thermoelectric voltage output. To achieve high sensitivity and oxidation resistance, some silicide, carbides, and conductive oxides have been developed as alternative electrodes for high temperature measurement, such as the working temperature of CrSi_2_-TaC TFTCs in a vacuum or inert gas going up to 1080 °C while the thermoelectric output remains stable. When it was in an oxidizing atmosphere, it failed at 455 °C. Meanwhile, CrSi_2_ can only work stably in an oxidizing environment at 670 °C; for more than 180 h, its sensitivity coefficient is 102 μV/°C [12,13]. MoSi_2_-TiSi_2_ carbide TFTCs was used to high temperature of 1200 °C. However, at high temperatures, SiO_2_ is formed due to oxygen entering the film, which leads the stability of the TFTCs to become worse due to the composition of the thin-film changes [14]. Compared to carbide and silicide thin film thermocouples, oxide ceramic TFTCs have more potential for high temperature stability and thermoelectric voltage output. Indium tin oxide (ITO) as a prevalent conductive oxide has been applied to TFTCs [15,16,17].

The basic principle of the thermocouple is based on the Seebeck effect, wherein two legs of the thermocouple have different Seebeck coefficients. Also, most of the metal and semiconductor thermoelectric materials are the same type. That is, the Seebeck coefficients of thermoelectric materials are all positive or negative. The Seebeck coefficients of tungsten-rhenium TFTCs are both positive, and the Seebeck coefficients of In_2_O_3_-ITO thermoelectric materials are negative. In order to increase the sensitivity of the TFTCs, one Seebeck coefficient of thermoelectric material is negative, while another, which is positive, is chosen. Therefore, some metal-oxide TFTCs are developed. Platinum (Pt) is a refractory precious metal with a low Seebeck coefficient and higher oxidation resistance, which can be used as a leg of TFTCs, such as Pt-ITO, Pt-ITON and Pt-In_2_O_3_ TFTCs [18,19,20]. During the heating cycle of 25–1200 °C, the Pt-ITO showed good stability; its Seebeck coefficient was up to 65.39 μV/°C [21]. Oxide (ITO) is annealed in a nitrogen atmosphere to improve the thermoelectricity stability of the Pt-ITON TFTCs. 

It is hoped that prepared TFTCs have strong high temperature resistance and a high thermoelectric voltage output. The tungsten-rhenium TFTCs have been reported for high temperature measurements of up to about 1500 °C [22,23,24]. Typical In_2_O_3_-ITO TFTCs have been reported which have shown a higher thermoelectric voltage output (173 mV at 1273 °C) and high temperature stability. To obtain high temperature resistance and high thermoelectric voltage output at the same time, WRe26 and In_2_O_3_ are chosen as a new combination of TFTCs based on the SI_3_N_4_ substrate. In this paper, the WRe26-In_2_O_3_ TFTCs were fabricated by RF magnetron sputtering, and the proprieties of In_2_O_3_ thin films and the thermoelectric voltage output of the TFTCs were analyzed under different annealing processes. The best annealing process was found to make the sensitivity of the WRe26-In_2_O_3_ TFTCs reach the expectation.

## 2. Theoretical Analysis

The principle of TFTCs are the same as the traditional wire thermocouples, which is based on the Seebeck effect [25,26]. When the hot junction is heated, and its temperature is *T*_1_. And the temperature of cold junction is *T*_0_. The thermoelectric potential can be measured at the cold junction of the thermocouples. The thermoelectric voltage of the thin film thermocouple is described as:

(1) $$E_{AB}=\int_{\theta_{0}}^{\theta }S_{AB}(T)dT=\int_{\theta_{0}}^{\theta }\left[S_{B}(T)-S_{A}(T)\right]dT$$

where the *S_AB_(T)* is the Seebeck coefficient of TFTC, *S_A_(T)* is the Seebeck coefficient of material A; *S_B_(T)* is the Seebeck coefficient of material B, *θ* is the temperature of hot junction; *θ*_0_ is temperature of cold junction. 

At the same time, the Seebeck coefficient of the conductive oxides are different from the metals. In_2_O_3_ is an N-type non-degenerate semiconductor material. The Seebeck coefficient of In_2_O_3_ is gave as:

(2) $$S\left(N_{D}\right)=-\frac{Ak}{e}-\frac{k}{e}In\left(\frac{{\left(2\pi m_{e}^{*}kT\right)}^{2/3}}{h^{3}N_{D}}\right)$$

where *S* is Seebeck coefficient, *K* is the Boltzmann constant, *h* is the Planck constant, *e* respects electronic charges; *N_D_* is carrier concentration, m_e_ is effective mass, *A* is a transport constant [27]. If additional oxygen enters the In_2_O_3_, it will affect the Seebeck coefficient of the In_2_O_3_. The conductive carriers of In_2_O_3_ mainly comes from the electrons released by the oxygen vacancy, and one oxygen vacancy contributes two electrons (Equation (3)) [28]. *V_O_* are doubly charged oxygen vacancies. When additional oxygen occupied the oxygen vacancy of the In_2_O_3_ film, it caused the carrier concentration in the In_2_O_3_ film to decrease while increasing the Seebeck coefficient of the In_2_O_3_.

(3) $$O_{o}^{x}\to 1/2O_{2}+V_{O}+2e^{-1}$$

**Figure 1:**
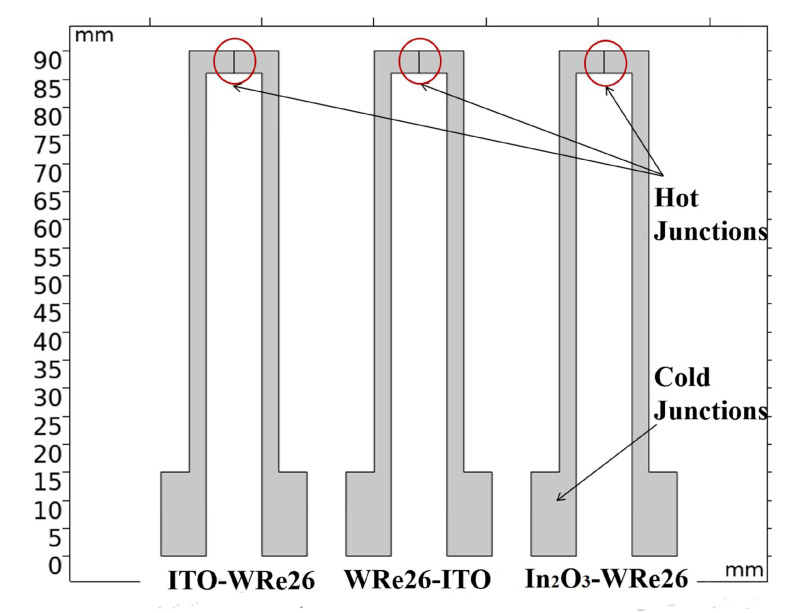
Simulation model of three thermocouple combinations.

**Figure 2:**
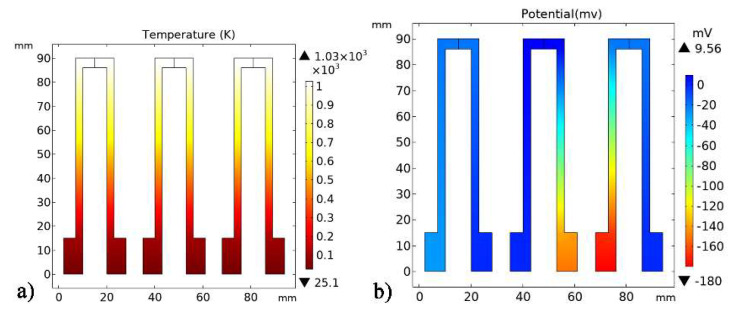
Simulation of three combinations. (**a**) The temperature difference distribution of thermocouples. (**b**) The thermoelectric voltage distribution.

**Figure 3:**
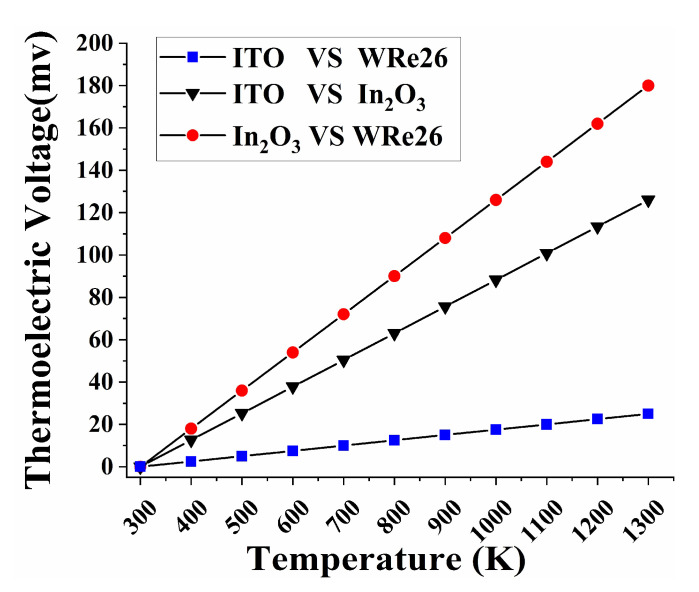
Thermoelectric voltage of three combinations simulation results.

To verify whether the thermoelectric voltage output of WRe26-In_2_O_3_ was better than the pure oxide combination (ITO-In_2_O_3_), thermoelectricity simulation of TFTCs with different thermoelectric material combinations was required. The thermoelectric characteristics of the ITO-WRe26, WRe26-In_2_O_3_ and ITO-In_2_O_3_ TFTCs were studied by using commercial software COMSOL to ensure the results of model analysis. Figure 1 shows the model of the three combinations of TFTCs. The single size of the TFTC is 30 mm × 90 mm. The area of hot junctions is 4 mm × 10 mm. In this analysis, the temperature of hot junctions was increased from 300 K to 1300 K, and the cold junctions were set to 293 K. The Finite Element Analysis results of temperature gradient and thermoelectric voltage distribution are presented in Figure 2. The maximum temperature of the hot junctions are 1300 K. Figure 3 shows the thermoelectric voltage output of TFTCs. According to the simulation results, thermoelectric output of WRe26-In_2_O_3_ is the biggest at 1300 K, which means the sensitivity coefficient of this combination is bigger than ITO-In_2_O_3_ in theory.

## 3. Experiment 

**Figure 4:**
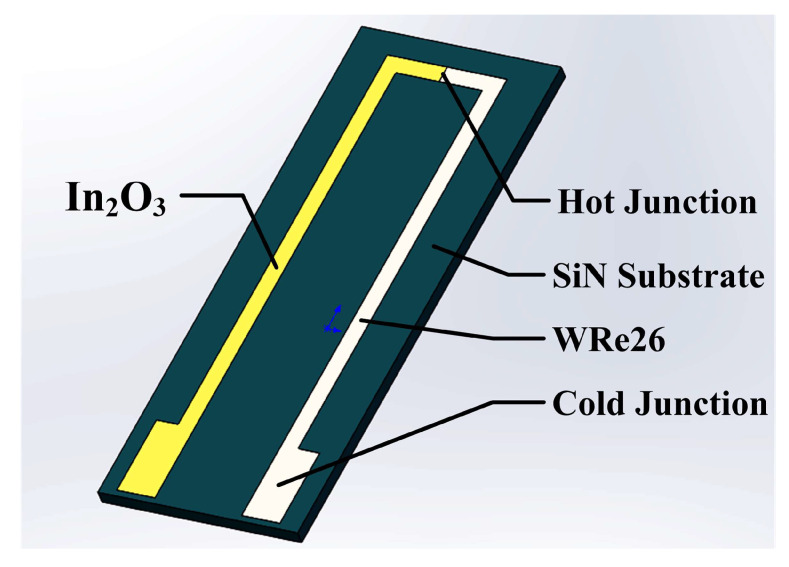
Structure of WRe26-In_2_O_3_ TFTCs.

In order to study the effect of different annealing on the thermoelectric voltage of TFTCs, In_2_O_3_ film samples and WRe26-In_2_O_3_ TFTCs were prepared by RF magnetron sputtering. RF Magnetron sputtering technology is widely used because of the good adhesion of the films on the substrate, good thickness uniformity and high film density [29,30,31]. High purity WRe26 and In_2_O_3_ Target (purity 99.999 wt.%, diameter: 101.6 mm, and thickness: 3 mm) were been used while the distance between target and substrate was 80 mm. In Figure 4, WRe26 and In_2_O_3_ films were deposited on the Si_3_N_4_ substrate. The mass size of Si_3_N_4_ substrate is 30 mm × 90 mm × 3 mm, and the TFTC is 8 mm × 70 mm × 2 um.

Table 1 shows the detail sputtering parameters of the TFTCs preparation. The order of deposition of the two legs of the TFTCs were especially important. The leg of WRe26-In_2_O_3_ TFTCs pattern was transferred by using photolithography. In_2_O_3_ films deposited by magnetron sputtering for 4 h. Then, In_2_O_3_ films were soaked in different annealing processes. After the TFTCs were cleaned up, the WRe26 films were sputtered for 90 min with a high power of 400 w. Finally, the Al_2_O_3_ protective layer was covered on the sensitive layer again. 

**Figure 5:**
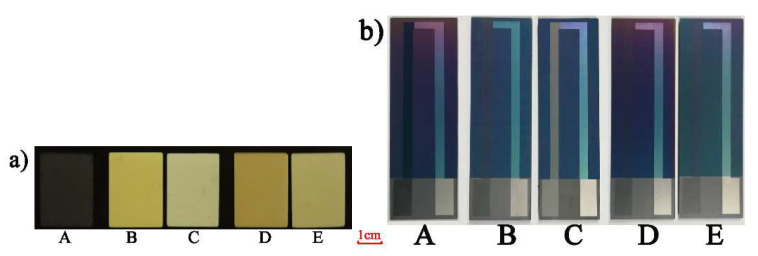
(**a**) Prepared In_2_O_3_ thin film samples at different annealing processes. (**b**) Prepared TFTCs under different annealing processes. (A: No annealing. B: 600 °Cin air for 2 h. C: 1000 °C in air for 2 h. D: 600 °C in vacuum for 2 h. E: 1000 °C in vacuum for 2 h.).

The In_2_O_3_ films samples at different annealing processes were presented in Figure 5a. The Al_2_O_3_ substrate was 14 mm × 20 mm × 1 mm. The color of film samples obviously changed under different annealing processes. The crystal structure of In_2_O_3_ at different annealing conditions was analyzed by X-ray diffraction (XRD), X-ray photoelectron spectroscopy (XPS) was used to characterize its chemical composition. Scanning electron microscopy (SEM) was used to observe the micro-morphology of In_2_O_3_ at different annealing conditions, and the WRe26-In_2_O_3_ TFTCs were prepared to find the best annealing process by thermoelectric voltage testing (Figure 5b). 

**Figure 6:**
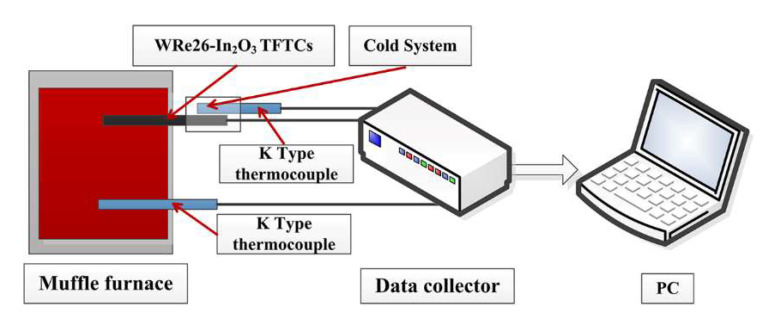
Thermoelectric test system of WRe26-In_2_O_3_ TFTCs.

Fabricated WRe26-In_2_O_3_ TFTCs were static tested in muffle furnace (LHT0820, Nabertherm, Lilienthal, Germany). As shown in Figure 6, one K-type thermocouples and WRe26-In_2_O_3_ TFTCs were placed in the muffle furnace to get the temperature of hot junctions. Another K-type was used to monitor the cold junctions. Cold junctions of the TFTCs were cooled by circulating cold water to maintain a big temperature gradient. Then thermoelectric voltage of K-type thermocouples and the WRe26-In_2_O_3_ TFTCs were recorded with a data collector (Hioki, LR8410-30, Nagano, Japan).

## 4. Result and Discussion 

**Figure 7:**
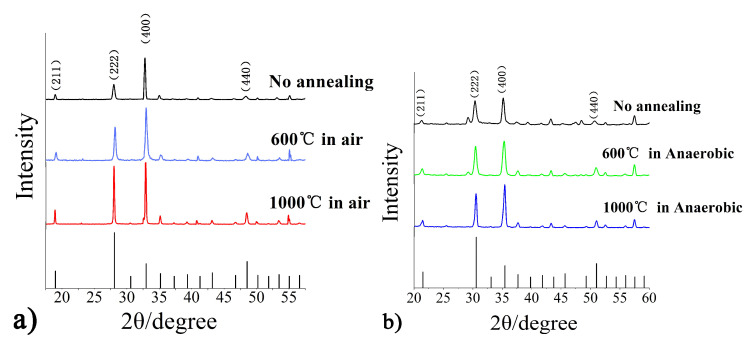
X-ray diffraction patterns of In_2_O_3_ film under different annealing processes, (**a**) Annealing process at different temperatures with air environment treatment, (**b**) Annealing process at different temperatures with vacuum treatment.

The X-ray diffraction (XRD) patterns of In_2_O_3_ film samples at different annealing process were presented in Figure 7. As shown in Figure 7a, the (222) peak of In_2_O_3_ is very small at no annealing. With increasing of air annealing temperature, the (222) and (400) peaks of In_2_O_3_ were promoted a great deal, especially the (222) peak increases in the air annealing at 1000 °C. This indicates that the preferred growth of the crystal plane are (222) and (400) crystal planes. In Figure 7b, it was obvious that each peak of In_2_O_3_ in XRD was nearly unchanged at the anaerobic annealing processes. 

**Figure 8:**
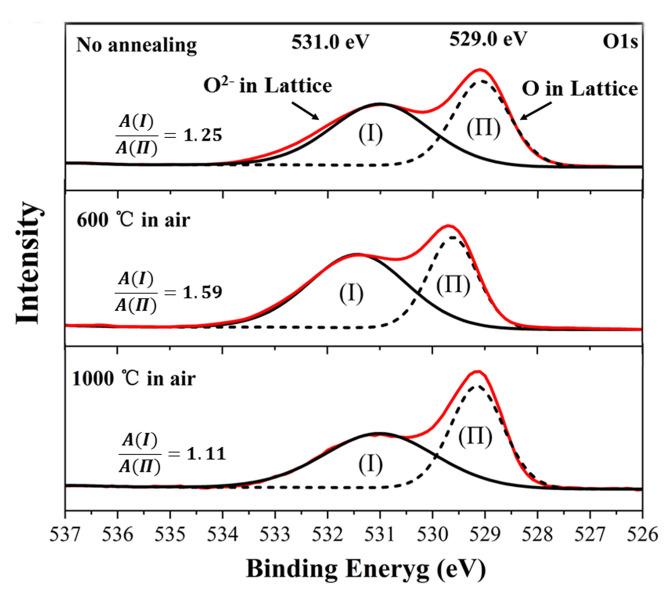
O1s photoelectron peaks of In_2_O_3_ in XPS at different annealing conditions.

XPS was used to analyze the oxygen element in In_2_O_3_ films at different annealing conditions. O 1s core energy spectrum of In_2_O_3_ films are shown in Figure 8. The O1s spectrum of In_2_O_3_ films has two peaks. The binding energy of 529 eV corresponds to the O element peak and binding energy of 531 eV corresponds to the O^2^^−^ element peak in In_2_O_3_ films. The area ratio under peak of O1s (I) and O1s (II) increased after 600 °C air annealing for 2 h. It is mainly because a large amount of oxygen in the air will not enter the film at a low temperature. Instead, oxygen escaped from the film to produce more oxygen vacancies and the O^2−^ element was increased. Then, In_2_O_3_ films recrystallized after annealing at 1000 °C for 2 h. More oxygen entered the In_2_O_3_ film, and oxygen vacancy defects were reduced, causing the carrier concentration of In_2_O_3_ to be reduced. 

**Figure 9:**
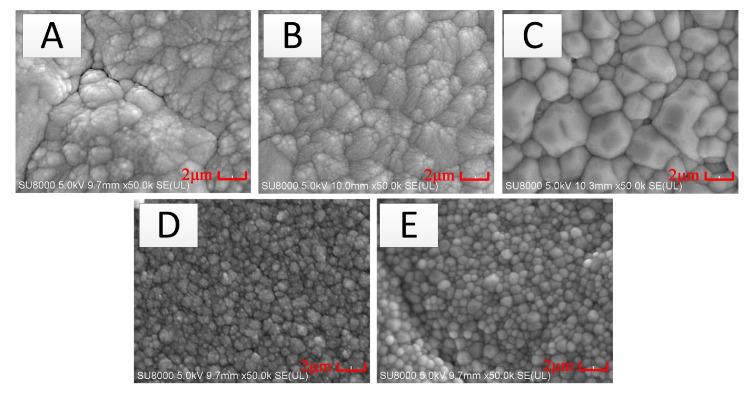
The SEM of In_2_O_3_ films under different annealing processes. (**A**: No annealing. **B**: 600 °C in air for 2 h. **C**: 1000 °C in air for 2 h. **D**: 600 °C with vacuum for 2 h. **E**: 1000 °C with vacuum for 2 h.).

Figure 9 exhibits the SEM of the In_2_O_3_ films under different annealing conditions. Compared to anaerobic and air annealing, as the temperature increased, the microstructures of In_2_O_3_ just became denser at anaerobic annealing. But the microstructures of In_2_O_3_ were changed significantly under air annealing. The organization grains of In_2_O_3_ became denser and larger, and the cellular crystals were formed at 1000 °C, implying that the oxygen entered the thin film structure at 1000 °C air annealing, and the oxygen occupied the oxygen vacancy of the In_2_O_3_ films, making the conductive electrons in the In_2_O_3_ film decrease rapidly according to the Equation (3). As a result, the Seebeck coefficient of In_2_O_3_ increased. 

**Figure 10:**
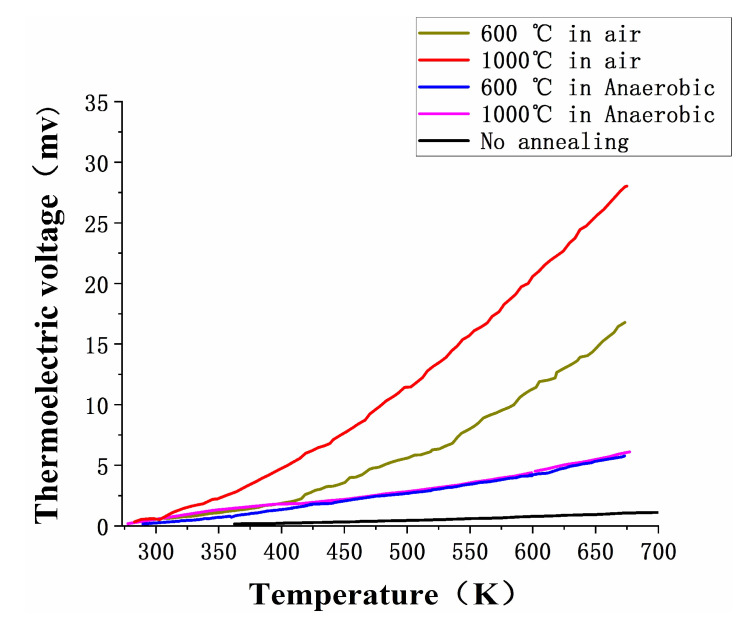
Thermoelectric voltage output of TFTCs.

To verify this phenomenon, Figure 10 shows the result of a static test of TFTCs from room temperature to 673 K at the different annealing process. It is obvious that the thermoelectric voltage was the smallest at no annealing. There were slight changes in the microstructures of In_2_O_3_ in 600 °C and 1000 °C anaerobic annealing. The thermoelectric voltage was significantly smaller than air annealing treatment. Thermoelectric voltage at 1000 °C air annealing was much bigger than 600 °C air annealing, which means the Seebeck coefficient of In_2_O_3_ films can be improved under air annealing processes, making the performance of WRe26-In_2_O_3_ TFTCs better. 

**Figure 11:**
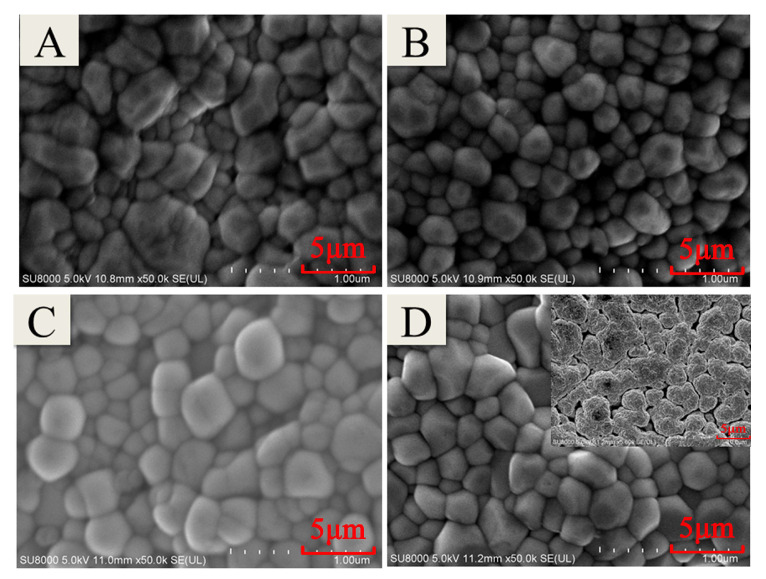
The SEM of In_2_O_3_ films under different annealing processes. (**A**: 1000 °C in air for 4 h. **B**: 1000 °C in air for 6 h. **C**: 1000 °C in air for 8 h. **D**: 1000 °C in air for 10 h.).

**Figure 12:**
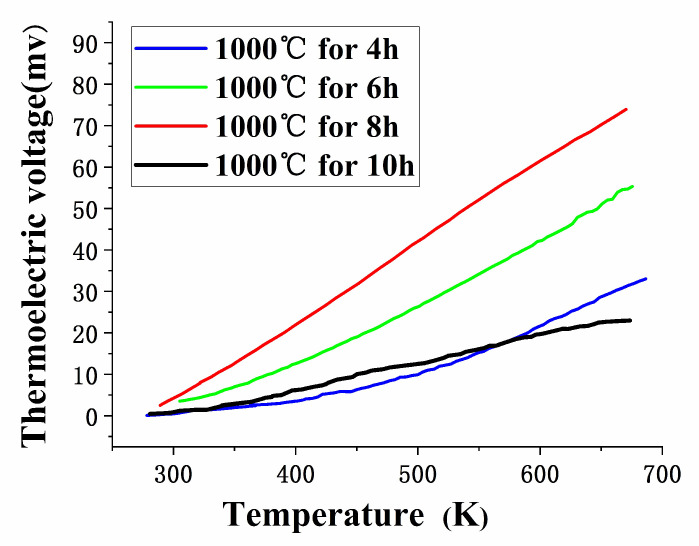
Thermoelectric voltage output of TFTCs.

In order to find the optimal annealing processes, the In_2_O_3_ films were annealed at 1000 °C for a longer time. As shown in Figure 11, with longer time in high temperature annealing, structure grains of In_2_O_3_ continued to grow and became more uniform, especially at 10 h. But from the test results of of WRe26-In_2_O_3_ TFTCs in Figure 12, it is observed that the thermoelectric voltage output is best at air annealing for 8 h. Thermoelectric voltage output for 10 h is smaller than that for 4 h. The reason was that voids appeared in the In_2_O_3_ films (Figure 11d), the grain boundaries of the In_2_O_3_ films structure become discontinuous, and the conductivity of the In_2_O_3_ films became poor during long duration annealing processes, although oxygen promoted the growth of tissue grains, leading to poor conductivity of the In_2_O_3_ film.

**Figure 13:**
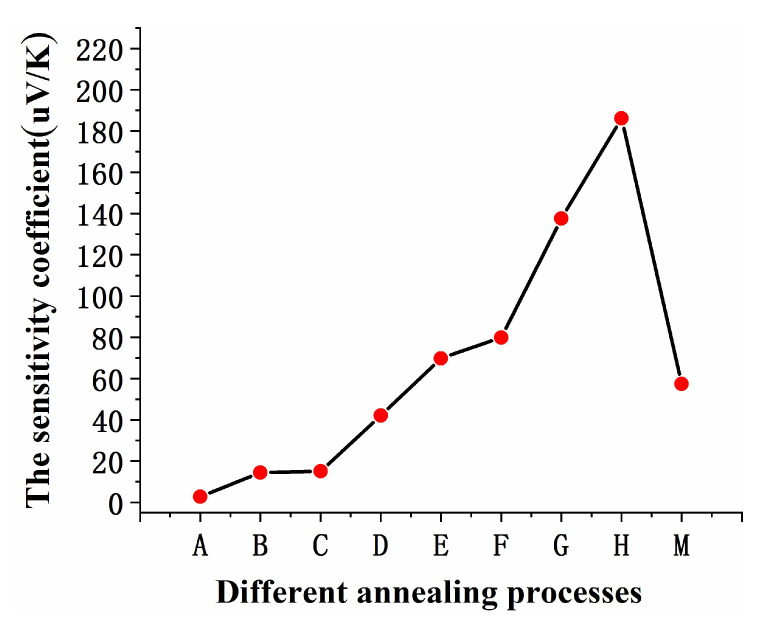
Average sensitivity coefficients of TFTCs at different annealing processes. (A: No annealing. B: 600 °C in air for 2 h. C: 1000 °C in air for 2 h. D: 600 °C with vacuum. E: 1000 °C with vacuum. F: 1000 °C in air for 4 h. G: 1000 °C in air for 6 h. H: 1000 °C in air for 8 h. M: 1000 °C in air for 10 h.).

The measured thermoelectric voltage depends on the difference between hot junction (*T_h_*) and cold junction (*T_c_*) and the Seebeck coefficient of the metal materials. The sensitivity coefficient (*S*) of thermocouples is given as:

(4) $$S=\frac{\Delta V}{\Delta T}=\frac{\Delta V}{T_{h}-T_{c}}$$

where the Δ*V* is the thermoelectric voltage difference between the WRe26 and In_2_O_3_. Figure 13 shows the average sensitivity (The temperature difference was 400K) of WRe26-In_2_O_3_ TFTCs at different annealing. The sensitivity coefficient of the TFTCs reached 186.1 μV/K at air annealing for 8 h.

**Figure 14:**
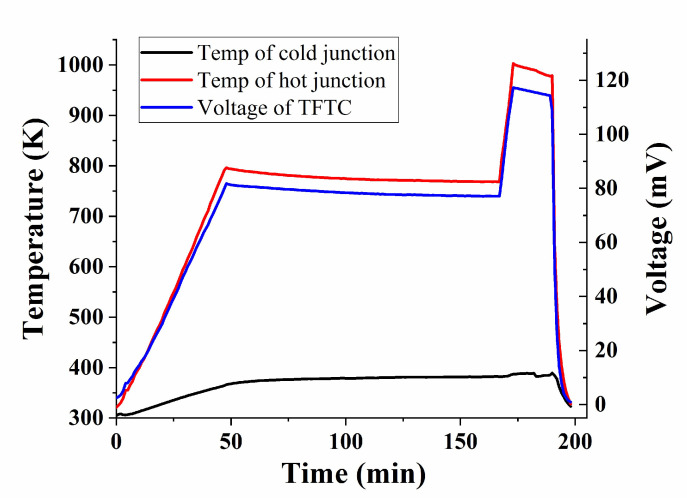
Thermoelectric voltage of WRe26-In_2_O_3_ TFTCs.

**Figure 15:**
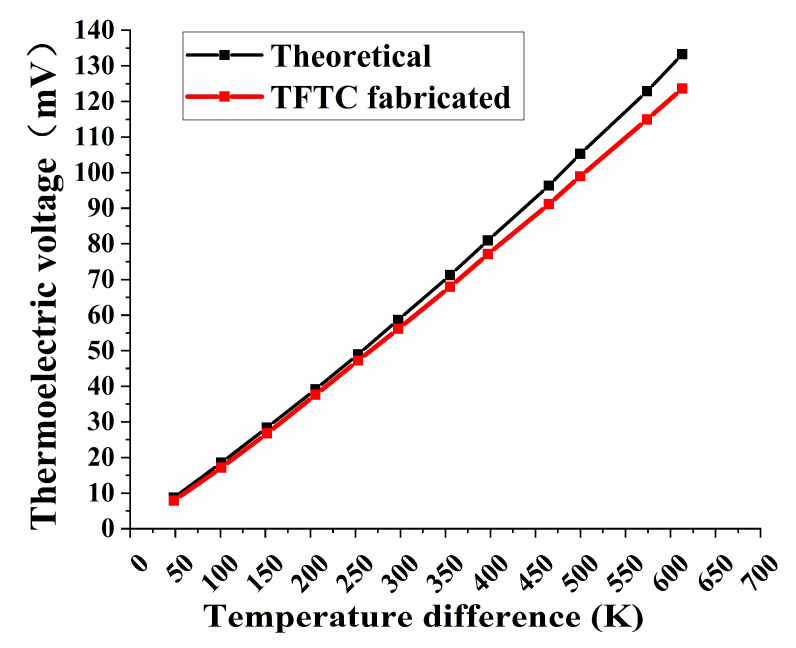
Static test thermoelectric voltage and theoretical curve of WRe26-In_2_O_3_ TFTCs.

Prepared WRe26-In_2_O_3_ TFTC was static calibrated in a high temperature after the optimal annealing process was determined. Figure 14 shows the temperature stability test of TFTC in muffle furnace. The WRe26-In_2_O_3_ TFTC and K-type thermocouples were raised from room temperature to 773 K and kept for two hours, and heated to 1000 K for twenty minutes. The heating rate was set at 10 °C/min. Then, TFTC was naturally cooled to room temperature. Figure 15 is a static thermoelectric voltage curve of WRe26-In_2_O_3_ TFTCs with the temperature difference up to 612.9 K. The hot junction of the thermocouple was 1000 K (the temperature of cold junction was 387.1 K), the thermoelectric voltage reached 123.6 mv. The average sensitivity coefficient was 201.6 μV/K. We have found the optimal annealing process at this magnetron sputtering process, but the Seebeck coefficient of In_2_O_3_ in the literature is about −200 μV/K, and the Seebeck coefficient of WRe26 is about 20 μV/K. So the sensitivity of the WRe26-In_2_O_3_ TFTCs is about 220 μV/K in theory. There was a little difference between the prepared TFTC and the theoretical thermoelectric output. This is mainly because the source of the In_2_O_3_ target was different, and so the Seebeck coefficient of In_2_O_3_ was also a little different. The Seebeck coefficient of In_2_O_3_ was highly affected by the quality In_2_O_3_ film.

## 5. Conclusions

In this study, a WRe26-In_2_O_3_ TFTC was reported. The WRe26-In_2_O_3_ TFTCs were successfully fabricated on the Si_3_N_4_ substrate by magnetron sputtering in order to improve the thermoelectric performance of the thermocouple. The properties of In_2_O_3_ films and the thermoelectric voltage properties of the WRe26-In_2_O_3_ TFTCs under different annealing processes were studied. The properties of In_2_O_3_ films at different annealing processes were analyzed by SEM, XRD, and XPS. The optimal annealing process of the TFTCs under this sputtering method was proposed. The WRe26-In_2_O_3_ TFTCs had ideal performance at the 1000 °C air annealing for 8 h. It was achieved that the average sensitivity of the WRe26-In_2_O_3_ TFTCs could reach 201.6 μV/K at a temperature difference of 612.9 K, which can maintain a stable output for 2 h at 773 K and 20 min for 1000 K.

## Figures and Tables

**Table 1:** Sputtering parameters of WRe26-In2O3 TFTCs.

Sputtering Parameters	WRe26	In_2_O_3_	Al_2_O_3_
Thickness (μm)	2	4	2
Power (W)	400	150	200
Presser (Pa)	1 × 10^−6^	1 × 10^−6^	5 × 10^−5^
Ar (sccm)	30	60	30
